# Inflammatory Stimulation Upregulates the Receptor Transporter Protein 4 (RTP4) in SIM-A9 Microglial Cells

**DOI:** 10.3390/ijms252413676

**Published:** 2024-12-21

**Authors:** Wakako Fujita, Yusuke Kuroiwa

**Affiliations:** 1 Laboratory of Pharmacotherapeutics, Faculty of Pharmacy, Juntendo University, Chiba 279-0013, Japan; 2 Department of Medical Pharmacology, Nagasaki University Graduate School of Biomedical Sciences, Nagasaki 852-8523, Japan; 3 Department of Pharmacology and Therapeutic Innovation, School of Pharmaceutical Sciences, Nagasaki University, Nagasaki 852-8521, Japan

**Keywords:** LPS, microglia, RTP4

## Abstract

The receptor transporter protein 4 (RTP4) is a receptor chaperone protein that targets class A G-protein coupled receptor (GPCR)s. Recently, it has been found to play a role in peripheral inflammatory regulation, as one of the interferon-stimulated genes (ISGs). However, the detailed role of RTP4 in response to inflammatory stress in the central nervous system has not yet been fully understood. While we have previously examined the role of RTP4 in the brain, particularly in neuronal cells, this study focuses on its role in microglial cells, immunoreactive cells in the brain that are involved in inflammation. For this, we examined the changes in the RTP4 levels in the microglial cells after exposure to inflammatory stress. We found that lipopolysaccharide (LPS) treatment (0.1~1 µg/mL, 24 h) significantly upregulated the *RTP4* mRNA levels in the microglial cell line, SIM-A9. Furthermore, the *interferon (IFN)-β* mRNA levels and extracellular levels of IFN-β were also increased by LPS treatment. This upregulation was reversed by treatment with neutralizing antibodies targeting either the interferon receptor (IFNR) or toll-like receptor 4 (TLR4), and with a TLR4 selective inhibitor, or a Janus kinase (JAK) inhibitor. On the other hand, the mitogen-activated protein kinase kinase (MEK) inhibitor, U0126, significantly enhanced the increase in RTP4 mRNA following LPS treatment, whereas the PKC inhibitor, calphostin C, had no effect. These findings suggest that in microglial cells, LPS-induced inflammatory stress activates TLR4, leading to the production of type I IFN, the activation of IFN receptor and JAK, and finally, the induction of *RTP4* gene expression. Based on these results, we speculate that RTP4 functions as an inflammation-responsive molecule in the brain. However, further research is needed to fully understand its role.

## 1. Introduction

Receptor transporter proteins (RTPs) are a class of receptor chaperone proteins that target Class A G-protein coupled receptor (GPCR)s, including taste and odor receptors, as well as opioid receptors [1,2,3]. There are four isotypes of RTP (RTP1, RTP2, RTP3, and RTP4) that are thought to function as chaperone proteins [2]. We recently reported that RTP4 plays an important role in the mechanisms involved in the development of antinociceptive tolerance to morphine [4]. We also found that morphine treatment upregulates *RTP4* in both microglia and neuronal cells [4]. The morphine-mediated upregulation of *RTP4* in microglial cells indicates that it may play a role in microglial function. However, to date, not much is known about the role of RTP4 in microglia.

Microglia, a key immune-responsive brain cell, has been shown to significantly contribute to the pathological changes that occur in the brain including neuroinflammation [5]. Moreover, recent studies show that the immune response in the brain plays a crucial role in the development and progression of various diseases, including diabetes, ischemic stress, and pain-related disorders [5]. Interestingly, recent research has highlighted the unique role of RTP4 as an inflammation regulatory protein [6]. These studies show that RTP4 is one of the interferon (IFN)-stimulated genes (ISGs) where RTP4 is upregulated by IFN receptor activation and regulates viral infections, including influenza, papilloma, yellow fever, norovirus, flavivirus, and CoV-2, among others [7,8]. Research has demonstrated that the N-terminal 3CXXC zinc finger domain (ZFD) region of RTP4 is crucial for antiviral activity [7]. RTP4 binds to replicating viral RNA via N-terminal 3CXXC ZFD and inhibits viral genome amplification [7]. Additionally, RTP4 may contribute to the suppression of type I IFN induction during the cyclic GMP-AMP synthase (cGAS)-stimulator of interferon genes (STING) pathway following pathogen infection, functioning as a negative regulator [9]. This phenomenon appears to be a compensatory or negative feedback mechanism wherein RTP4 is initially induced by type I IFN and specifically binds to TANK-binding kinase 1 (TBK1), resulting in inhibition of type I IFN production [10]. Consequently, it is hypothesized that RTP4 functions as an inflammatory regulator of the immune system. Although the precise role of RTP4 in the inflammatory mechanism has not been fully elucidated, the observation that RTP4 is upregulated in peripheral tissues under various inflammatory conditions, such as colitis [11] and allergic asthma [12], suggests its physiological significance in inflammatory processes.

Conversely, RTP4 has been identified as a receptor chaperone protein that contributes to the maturation or translocation of class A GPCR such as taste, odorant, and opioid receptors [1,2,3], as previously described. Although the N-terminal region of RTP4 has been demonstrated to be involved in its distinct function as a chaperone molecule [1], the potential crosstalk or link between RTP4 or related signaling partners as immune regulatory molecules and chaperone proteins remains to be elucidated.

Anatomically, RTP4 was found to play important roles in peripheral immune cells, such as macrophages, in response to infections [7,9,10,13]. It is worth noting that *RTP4* is primarily expressed in macrophages throughout the body, as shown by database analysis [14]. Thus, it is crucial to focus on the role of RTP4 in central immune cells particularly in the context of microglia, immunoreactive cells in the brain, and neuroinflammation. In this study, we investigate the cellular mechanisms underlying the regulation of *RTP4* gene expression in microglia. We use lipopolysaccharide (LPS) stimulation to induce immune or inflammatory stress and examine its effect on RTP4 expression as well as inflammation-related genes in microglia.

## 2. Results

We treated SIM-A9 microglial cells with LPS to induce inflammatory stress and assessed the changes in mRNA levels of *RTP4* and inflammation-related genes by quantitative PCR analysis. We also determined the effect of neutralizing antibodies targeting selective receptors or of selective kinase inhibitors to reveal the downstream mechanisms involved in RTP4 gene induction.

### 2.1. Changes in RTP4 mRNA Levels After LPS Treatment in SIM-A9 Microglial Cell Line

**Figure 1:**
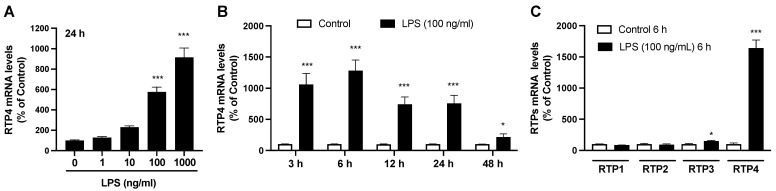
Changes in *RTP4* mRNA levels after LPS stimulation in SIM-A9 microglial cell line. Cells were treated with LPS (100 ng/mL) for 24 h (**A**), 6 h (**C**) or for indicated periods (**B**) and then collected to perform RT-qPCR analysis by using selective primers targeting *GAPDH* (internal control) or *RTP*s. Control cells were treated with a vehicle instead of LPS for the indicated periods. Data are the mean ± S.E.M. n = 10 (**A**), n = 7 (**B**), n = 4 (**C**), * *p* < 0.05, *** *p* < 0.0001, vs. control (without LPS), one-way ANOVA and Tukey’s multiple comparison test (**A**), multiple unpaired *t*-test (**B**,**C**).

Quantitative PCR analysis shows that 24 h treatment with LPS significantly increases *RTP4* mRNA levels in a concentration dependent manner (Figure 1A). Time course studies show robust *RTP4* upregulation from 3 to 24 h treatment with LPS (Figure 1B). Interestingly, we find that LPS treatment does not increase the mRNA levels of other *RTP*s (i.e., *RTP1* and *RTP2*) or at least not significantly, but slightly increases *RTP3*, indicating that it specifically and robustly upregulates *RTP4* in these cells (Figure 1C).

### 2.2. Alterations in RTP4 Expression Following LPS Treatment in SIM-A9 Microglial Cell Line: A Study of Immunofluorescence Analysis

**Figure 2:**
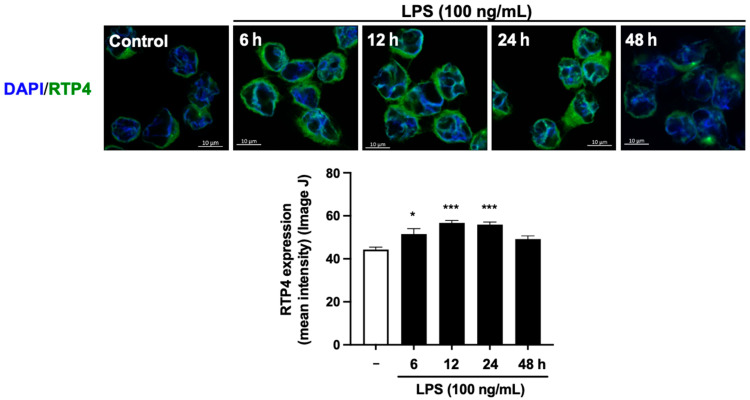
The effect of LPS stimulation on the expression levels of RTP4 determined by immunofluorescent analysis. SIM-A9 microglial cells were treated without or with LPS (100 ng/mL) for the indicated periods and immunofluorescent analysis performed as described in Materials and Methods. Control cells (−) were treated with vehicle instead of LPS for 24 h. Scale bar is 10 micrometer. Data are the mean ± S.E.M. n = 144 (Control), n = 53 (LPS 6 h), n = 138 (LPS 12 h), n = 130 (LPS 24 h), n = 100 (LPS 48 h) ’n’ represents the total number of cells from 3 independent experiments. * *p* < 0.05, *** *p* < 0.0001, vs. control, one-way ANOVA and Tukey’s multiple comparison test.

We used immunofluorescence analysis to examine cellular RTP4 expression in SIM-A9 microglial cells. We find that compared to control cells the signal for RTP4 was stronger in the cells treated with LPS for 6, 12, and 24 h but not after 48 h treatment (Figure 2, upper panel). The quantification of these data supports a marked increase in the immunoreactive signal for RTP4 following LPS treatment for 6, 12, and 24 h (Figure 2, lower panel). Together, quantitative PCR and immunofluorescence analysis indicate that *RTP4* mRNA and protein levels are temporarily elevated following LPS treatment (Figure 2).

### 2.3. Alterations in mRNA Levels of Inflammatory Mediators Following LPS Treatment in the SIM-A9 Microglial Cell Line

**Figure 3:**
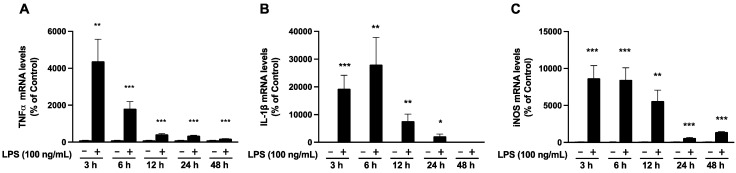
Changes in *TNFα* (**A**), *IL-1β* (**B**), and *iNOS* (**C**) mRNA levels after LPS treatment. SIM-A9 microglial cells were treated with LPS (100 ng/mL) for the indicated periods and then collected to perform RT-qPCR analysis using selective primers targeting *GAPDH* (internal control), *TNFα, IL-1β* or *iNOS*. Control cells (−) were treated with a vehicle instead of LPS for indicated periods. Data are the mean ± S.E.M. n = 12 (3 and 12 h); n = 16 (6 and 24 h); n = 10 (48 h), * *p* < 0.05, ** *p* < 0.001, *** *p* < 0.0001, vs. control, multiple unpaired t-test.

We evaluated changes in the mRNA levels of several pro-inflammatory cytokines, including *TNFα*, *IL-1β*, and *iNOS*, and found that their levels were significantly elevated in the SIM-A9 microglial cells within 48 h of LPS stimulation. The *TNFα* mRNA levels displayed a significant increase at every time point post-LPS treatment, although a gradual decrease was observed after 6 h of LPS stimulation (Figure 3A). The upregulation of *IL-1β* was detected upto 6 h post-LPS treatment, after which it gradually decreased but was significant up to 24 h (Figure 3B). Interestingly, the *iNOS* mRNA levels also showed higher levels up to 6 h post-LPS treatment, after which it gradually decreased, but was significant up to 48 h (Figure 3C). 

### 2.4. Alterations in Interferon mRNA Levels Following LPS Treatment in SIM-A9 Microglial Cell Line

**Figure 4:**
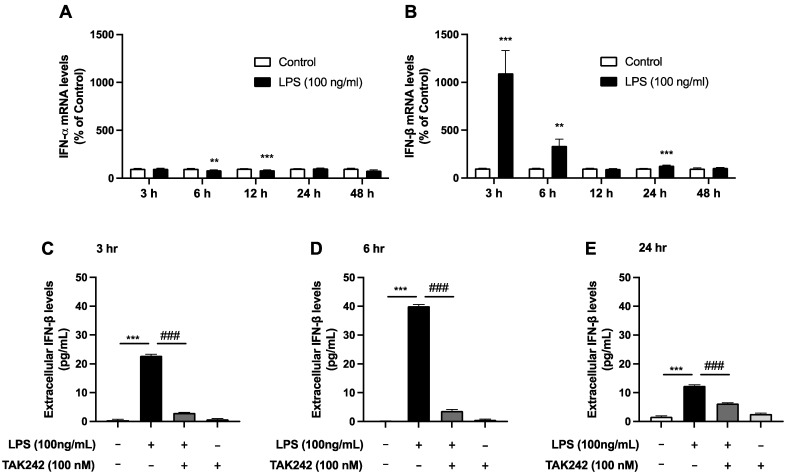
Changes in *IFN-α* (**A**) and *IFN-β* (**B**) mRNA levels after LPS treatment. SIM-A9 microglial cells were treated with LPS (100 ng/mL) for the indicated periods and collected for RT-qPCR analysis as described in Methods using selective primers targeting *GAPDH* (internal control), *IFN-α*, or *IFN-β*. Control cells were treated with a vehicle instead of LPS for indicated periods. Data are the mean ± S.E.M. n = 12 (3, 12 h), n = 16 (6, 24 h), n = 10 (48 h), ** *p* < 0.001, *** *p* < 0.0001, vs. control, one-way ANOVA, and Tukey’s multiple comparison test. Effect of TAK242, a TLR4 inhibitor, on the increase in extracellular IFN-β levels after LPS treatment (**C**–**E**). SIM-A9 microglial cells were treated with LPS (100 ng/mL) for the indicated periods and the culture medium was collected for ELISA analysis as described in Methods. Data are the mean ± S.E.M. n = 5–6, *** *p* < 0.0001, vs. control, ### *p* < 0.0001, vs. LPS alone. Multiple unpaired *t*-test (**A**,**B**), one-way ANOVA and Turkey’s multiple comparison test (**C**–**E**).

Next, we examined the expression of type 1 interferon (IFN), including *IFN-α* and *IFN-β*, following LPS treatment. In the case of *IFN-α*, we did not detect a significant increase in the mRNA levels at any time point following LPS treatment compared to vehicle- treated controls; rather, a slight but significant decrease was observed at 6 and 12 h after LPS treatment (Figure 4A). For *IFN-β*, we detected significant increases in the mRNA levels after 3 h and 6 h LPS treatment, with levels returning to those of vehicle-treated control after 12 h LPS treatment, while a slight but significant increase was observed at 24 h (Figure 4B). Furthermore, we observed that IFN-β was significantly detected in the culture medium at 3, 6, and 24 h post-LPS treatment, with the most substantial increase noted at 6 h following LPS administration (Figure 4C–E). The LPS-induced elevation of extracellular IFN-β was significantly attenuated by TAK242, a selective TLR4 inhibitor (Figure 4C–E).

### 2.5. The Effect of Neutralizing Antibody Targeting TLR4 or IFN Receptor (IFNR) and of Inhibitors of TLR4 or IFNR Signaling Molecules on LPS-Induced Upregulation of RTP4 Levels

**Figure 5:**
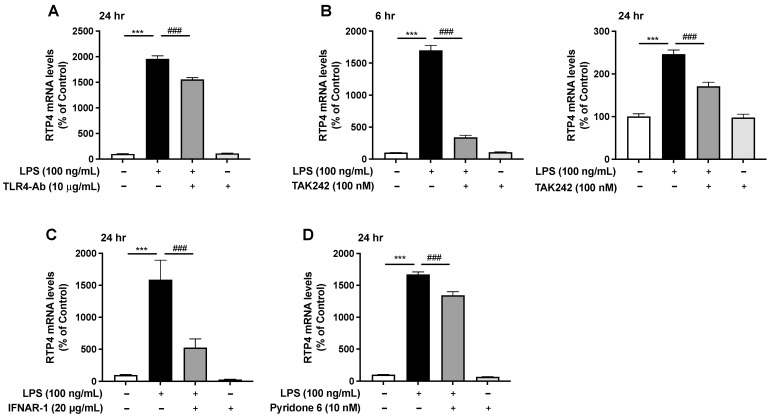
The effect of neutralizing antibody targeting TLR4 (**A**), selective inhibitor of TLR4 (TAK242) (**B**), neutralizing antibody targeting IFNR (IFNAR-1) (**C**) and inhibitor of JAK (Pyridone 6) (**D**) on LPS-induced upregulation of *RTP4* mRNA levels. SIM-A9 microglial cells were pretreated with TLR4 antibody (10 µg/mL), TAK242 (100 nM), IFNR antibody (20 ug/mL) or Pyridone 6 at indicated concentrations for 30 min before the LPS (100 ng/mL) or vehicle treatment for 6 h or 24 h which cells were collected and subjected to RT-qPCR analyses using primers that target *GAPDH* and *RTP4*. Control cells were pretreated with medium instead of antibody or inhibitor and treated with vehicle instead of LPS for 24 h. Data are the mean ± S.E.M. n = 3–7 (**A**), n = 5–6 (**B**), n = 7 (**C**), n = 4 (**D**). *** *p* < 0.0001, vs. control, ### *p* < 0.0001, vs. LPS alone. One-way ANOVA and Turkey’s multiple comparison test.

Next, we examined the effect of a neutralizing antibody targeting TLR4 on the LPS-induced upregulation of *RTP4* mRNA levels. We found that while on its own this antibody did not have any effect on the *RTP4* mRNA levels, it caused a small but significant decrease in the LPS-induced upregulation of the *RTP4* mRNA levels (Figure 5A). In this study, we treated the cells with LPS and inhibitors for 24 h to confirm the effect of the inhibitors for up to 24 h. In addition, we confirmed that TAK242, a selective TLR4 inhibitor, significantly suppressed the increase in *RTP4* mRNA levels after 6 and 24 h LPS treatment (Figure 5B). Next, we examined the effect of a neutralizing antibody targeting the INF receptor (INFR) on the LPS-induced upregulation of *RTP4* mRNA levels. We found that while on its own this antibody did not have any effect on *RTP4* mRNA levels, it caused a robust and significant inhibition of LPS-induced upregulation of *RTP4* mRNA levels (Figure 5C). Since INFR can induce signaling via the JAK/STAT pathway [15], we examined the effect of a pan-JAK inhibitor (Pyridone 6) on the LPS-induced upregulation of *RTP4* mRNA levels. We found that while on its own Pyridone 6 did not have any effect on *RTP4* mRNA levels, it caused a small but significant inhibition in the LPS-mediated upregulation of *RTP4* mRNA levels (Figure 5D).

### 2.6. The Effect of Inhibitors of TLR4 Signaling Molecules on LPS-Induced Upregulation of RTP4 Levels

**Figure 6:**
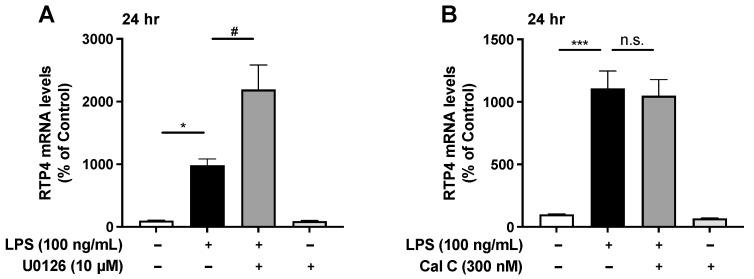
The effect of inhibitors of MAPK kinase (**A**) and PKC (**B**), TLR4 downstream signaling molecules, on LPS-induced upregulation of *RTP4* mRNA levels. SIM-A9 microglial cells were pretreated with inhibitors at indicated concentrations for 30 min before the LPS (100 ng/mL) or vehicle treatment for 24 h after which cells were collected and subjected to RT-qPCR analyses using primers that target *GAPDH* and *RTP4*. Control cells were pretreated with medium instead of inhibitor and treated with vehicle instead of LPS for 24 h. Data are mean ± S.E.M. n = 8, * *p* < 0.05, *** *p* < 0.0001, vs. control, # *p* < 0.05, vs. LPS alone, n.s., not significant, one-way ANOVA and Turkey’s multiple comparison test.

Since studies have shown that treatment with the MAPK kinase inhibitor (U0126) or with the PKC inhibitors block TLR4-mediated signaling [16,17] we examined the effect of these inhibitors on the LPS-induced upregulation of *RTP4* mRNA levels. Notably, we found that while on its own U0126 did not have any effect on *RTP4* mRNA levels, it significantly enhanced the upregulation of the *RTP4* mRNA levels induced by LPS (Figure 6A). In the case of the pan-PKC inhibitor, calphostin C, we found that it had no effect on the *RTP4* mRNA levels when administered alone or in combination with LPS (Figure 6B).

## 3. Discussion

**Figure 7:**
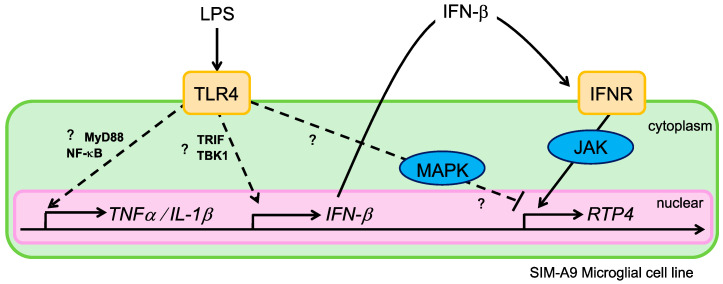
Illustration of the TLR4 signaling pathways and the mechanism of *RTP4* induction under LPS stimulation. Following LPS stimulation, TLR4 activation leads to IFN-β production in microglial cells. IFN-β is subsequently released into the extracellular compartment and transactivates IFNR, resulting in *RTP4* production. ‘?’ and the dashed line indicates a pathway that has not been elucidated in this study and is thus a hypothesis here.

In this study, we aimed to investigate alterations in *RTP4* mRNA levels following LPS treatment in SIM-A9 microglial cells. Our data are consistent with the idea that LPS stimulation leads to the upregulation of *RTP4* mRNA levels through TLR4 and IFNR, and subsequently through the intracellular signal molecule JAK. In addition to *RTP4*, we confirmed that inflammatory cytokines such as *TFNα*, *IL-1β*, *iNOS*, and *IFN-β* were transiently upregulated following LPS stimulation (Figure 3). These findings suggest that LPS stimulation induces an inflammatory response in a SIM-A9 microglial cell line. It is worth noting that the peak changes in the mRNA levels of each molecule including *RTP4* occurred within a similar time frame (approximately 3–6 h) suggesting that they may have been induced concurrently and/or independently upon activation of TLR4 [18]. In addition, our data clearly indicates that the transactivation of IFNR via extracellular IFN-β plays a role in LPS-mediated *RTP4* gene induction (Figure 7).

### 3.1. Characteristics of SIM-A9 Microglial Cells as an In Vitro Model of Endogenous Microglia

Although it is important to demonstrate or determine the pathophysiological role of RTP4 in primary cultured microglia or by using in vivo studies, here we used the SIM-A9 microglial cell line as an in vitro model as the first step of the series of studies.

From the point of view of similarity, we found that the expression pattern of the RTP isotypes in the SIM-A9 microglial cell line was similar to that of mouse brain regions, as well as in the Neuro2a neuronal cell line (Supplementary Figure S1). Furthermore, our recent study showed a similar RTP expression pattern in the spinal cord [19]. These data suggest that *RTP4* is consistently the most highly expressed isotype in cell lines, as well as in the mouse brain.

Furthermore, the SIM-A9 microglial cells used in this study are known as the ones that developed a novel, spontaneously immortalized microglia-A9 cell line (SIM-A9) from a primary glial culture of postnatal murine cerebral cortices [20,21]. Genetically or pharmacologically non-transformed SIM-A9 microglial cells exhibit endogenous microglial phenotypes, such as the secretion of inflammatory mediators, including TNF-α, along with phagocytic activity similar to cultured primary microglia in response to LPS, which are retained over 40 passages [20]. This distinguishes phenotypes from retrovirally transformed immortalized microglia such as BV-2 and N9 derived from rat and mouse where the viral transformation might alter the microglial phenotype and thus possess only short-term microglial properties [20,22,23]. 

Importantly, it has already been validated that SIM-A9 microglial cells retain typical microglial characteristics after cloning, and respond to inflammatory stimuli in a similar manner to primary microglia, especially with regard to phagocytic activity and pro-inflammatory signaling in response to LPS stimulation [20]. Furthermore, SIM-A9 microglial cells have recently been used as an in vitro model of activated microglia in the context of neuropathic pain [21], suggesting the reliability of using this cell line as the first step in in vitro pathophysiological analysis, which could be further developed for in vivo studies.

### 3.2. The TLR4-Mediated RTP4 Induction in SIM-A9 Microglial Cells

Since we found a clear induction of *RTP4* mRNA and protein levels by LPS treatment, we next focused on the trigger of this phenomenon. The differences in the expression changes in RTP4 between protein and mRNA might be due to differences in analytical sensitivity (Figure 1 and Figure 2). Furthermore, these findings should be validated in future studies using primary cultured microglia and in vivo experiments as described above.

Given that we utilized LPS as the inflammatory stimulus, we initially focused on the cell surface receptor for LPS. TLR4 is one of the members of the TLR family, a group of pattern recognition receptors known as LPS receptors [24,25] and critical for an effective innate immune response [26,27,28]. TLR4 is the most relevant receptor targeted by LPS, and we found that the neutralizing antibody targeted TLR4 partially but significantly suppressed the LPS-induced *RTP4* induction (Figure 5A). The partial inhibition might be due to the antibody concentration used in this study, which is the highest concentration we could use due to resource limitations. Significantly, the observation that TAK242, a selective TLR4 inhibitor, completely suppressed the upregulation of *RTP4* mRNA levels at 6 and 24 h after LPS treatment (Figure 5B) provides strong evidence for the involvement of TLR4 in the mechanism of *RTP4* induction following LPS treatment.

### 3.3. The Interferon Receptor-Mediated Mechanism of RTP4 Induction in SIM-A9 Microglial Cells

Several previous reports have suggested that *RTP4* can be induced by interferon [7,29]. This is consistent with our data in this study. Significantly, the findings of this investigation indicate that LPS stimulation induces the release of IFN-β via TLR4, leading to the activation of IFNR and its downstream signaling molecules, such as JAK, thereby resulting in *RTP4* induction by LPS. To elucidate the involvement of JAK, this study employed a pan-JAK inhibitor and observed its effect on LPS-mediated upregulation of *RTP4* mRNA levels. Further studies probing specific subtypes of JAK, such as JAK2, could reveal their involvement in LPS effects in microglia [30]. It is interesting to note that type-I-interferon responsive microglia have been reported to play a critical role in cortical development and sensorimotor function [31]. Given the potential physiological significance of interferon-responsive molecules, including RTP4, it is important to assess the extent to which RTP4 in microglia plays a role in normal brain function and pathology.

### 3.4. Inverse Regulation of RTP4 Gene Expression via MAPK Signaling Pathway

The current study has revealed that LPS-mediated *RTP4* gene expression may be negatively regulated by the MAPK signaling pathway (Figure 6A) since it is enhanced by treatment with the MAPK inhibitor, U0126. Since we did not directly determine the phosphorylation levels of ERK after LPS stimulation using Western blot analysis, it is unclear whether the “activation or phosphorylation” of ERK has occurred before or is concomitant with the upregulation of *RTP4* mRNA levels. Other studies have demonstrated that LPS stimulation activates ERK in SIM-A9 microglial cells [32]. Thus, we assumed that this activation might also have occurred in this study. Future studies are needed to elucidate the activation of ERK.

These results imply the existence of a suppressive or silencing mechanism for *RTP4* gene induction in SIM-A9 microglial cells. It is a reasonable physiological idea that cells are primarily regulated through both positive and negative signaling mechanisms [33]. Although the mechanisms involved in the suppression of *RTP4* gene expression have yet to be elucidated, it can be speculated that the mechanisms of gene silencing (such as transcriptional repression, epigenetic modulation, including DNA methylation or histone modification) that are under the MAPK signaling regulation may be involved [34]. Further investigation is needed to determine the involvement of these pathways. Interestingly, although LPS has been shown to activate most of the PKC isoforms expressed in macrophages [35,36,37,38], in this study, we find that PKC inhibition did not affect LPS-mediated *RTP4* mRNA upregulation supporting the notion that the PKC-mediated pathway may not contribute to the effects of LPS on *RTP4* gene expression (Figure 6B).

### 3.5. The TLR4 Signal Pathways and the Mechanism of RTP4 Induction Under LPS Stimulation

The TLR4-mediated mechanism is known to include canonical (i.e., MyD88-nuclear factor κB (NF-κB) dependent) and non-canonical (TRIF-dependent) signaling pathways [39,40]. LPS-induced TLR4/TRIF-dependent signaling results in type I IFN production, whereas MyD88-NF-kB signaling leads to the expression of pro-inflammatory genes, including *TNFα* and *IL-1β* [41,42]. Based on the aforementioned findings, a proposed mechanism for *RTP4* induction is illustrated in Figure 7. It is hypothesized that RTP4 is a molecule responsive to IFN stimulation via IFNR and is thus regulated by the non-canonical signaling pathway of TLR4, while remaining independent of TNFα / IL-1β production in this study. Moreover, this investigation has demonstrated that the MAPK-mediated pathway may serve a silencing or compensatory role in *RTP4* production, although further research is required to elucidate this relationship.

### 3.6. The Role of RTP4 in the Inflammatory Response

Another observation of this study is that *RTP4* is the only isotype of the RTP family that is significantly induced by LPS (Figure 1C). From a structural perspective, not much is known about the domains of RTP4 involved in inflammatory responses. RTP4 is a 247 amino acid protein composed of an amino-terminal (N-terminal) zinc finger domain (ZFD), an intrinsic disordered variable region, and a single carboxy-terminal (C-terminal) transmembrane domain (TM). The ZFD has been demonstrated to be a crucial region in the regulation of viral RNA replication [43]). Additionally, the presence of a 3CxxC motif at a crucial amino acid site (Cysteine 95) in the ZFD of RTP4 has been identified as essential for its antiviral function [44]. Thus, studies are needed to evaluate the crucial structural features and specific domains of RTP4 that are involved in inflammatory responses.

### 3.7. Future Perspective

Studies are needed to evaluate in detail the specific role of RTP4 in microglial cells in response to inflammatory stress, given that RTP4 is the sole RTP isotype that is induced by LPS treatment. In addition, it would be suggested that these findings should be reproduced in primary cultured microglia and in vivo experiments in a future study. Since microglia can exhibit both pro-inflammatory (M1) and anti-inflammatory (M2) phenotypes, the induction of RTP4 might play a role in determining the microglial phenotype. While RTP4 in macrophages has been reported to function as an anti-immune response molecule by influencing interferon expression, its role in microglia remains unclear [5]. Furthermore, since RTP4 has been associated with multiple functions, including improving pregnancy outcomes, serving as a marker of disease incidence, exhibiting antiviral activity, and acting as a receptor chaperone protein [7,8,45,46], it would be of interest if studies investigated whether RTP4 shares a common mechanism across these various functions.

## 4. Materials and Methods

### 4.1. Chemicals

The following reagents were used: Lipopolyssaccharide (LPS) (Sigma-Aldrich, St. Louis, MO, USA), Calphostin C, an inhibitor of protein kinase C (PKC) (Tocris, Bio-Techne Japan, Tokyo, Japan), U0126, a potent, selective inhibitor of MEK1 and 2 (Tocris, Bio-Techne Japan, Tokyo, Japan), Pyridone 6, a potent pan-JAK inhibitor (Tocris, Bio-Techne Japan, Tokyo, Japan), TAK242, a selective Toll-like receptor 4 (TLR4) inhibitor (Merk, Japan). Monoclonal IFNAR-1 (IFN-Ab, Bio X Cell, Lebanon, NH, USA) and TLR4/MD-2 Complex Monoclonal Antibody (TLR4-Ab, Thermo Fisher Scientific, Waltham, MA, USA) were diluted with culture medium as described in the figure legends.

### 4.2. SIM-A9 Microglial Cell Culture and Treatment

SIM-A9 microglial cells (ATCC, Manassas, VA, USA) were grown in complete growth medium (DMEM (Wako, Osaka, Japan) with 10% Fetal Bovine Serum (Thermo Fisher Scientific), 5% Horse Serum (Thermo Fisher Scientific), and 1% Penicillin-Streptomycin (nacalai tesque, Kyoto, Japan)) at 37 °C and under 5% CO_2_. The SIM-A9 microglial cells (8 × 10^4^) in culture medium were seeded into poly-DL-lysine (Sigma-Aldrich) coated 24-well plates one day before the experiment. The next day, the media was replaced with 500 µL of the complete growth medium containing LPS or vehicle (0.01% PBS in culture media) at the final concentration of 1 to 1000 ng/mL and incubated for 3 to 48 h at 37 °C under 5% CO_2_. In one set of experiments, cells were pretreated with inhibitors or neutralizing antibody for 30 min followed by co-treatment with LPS and reagents for 24 h. After ligand treatment, the SIM-A9 microglial cells were washed once with cold phosphate-buffered saline (PBS) (Wako) and then collected with 300 µL of cold RLT buffer (QIAGEN, Hilden, Germany). The cell lysates were transferred into an RNase free centrifuge tube and stored at −80 °C until they were further processed for RT-qPCR. At the same periods, the supernatant was collected and centrifuged at 1000× *g* at 4 °C for 10 min. The supernatant was collected and stored at −80 °C until they were further processed for ELISA.

### 4.3. RT-qPCR

RT-qPCR was performed as described previously [47]. We extracted RNA-containing aqueous solution using the TRIzol reagent according to the manufacturer’s protocol (Thermo Fisher Scientific Inc.) before total RNA purification. The total RNA was purified using an RNeasy Mini kit (QIAGEN Inc., Germantown, MD, USA) according to the manufacturer’s protocol. The cDNA was synthesized using the PrimeScriptTM RT Master Mix (Takara, Shiga, Japan) according to the manufacturer’s protocol. Real-time PCR was performed using the Power SYBR Green qPCR Master Mix (Applied Biosystems, Foster City, CA, USA). The PCR template source was 4 µL of 10-times diluted first-strand cDNA. Amplification was performed with an ABI PRISM 7900HT sequence detection system (Applied Biosystems). After an initial denaturation step at 95 °C for 10 min, amplification was performed using 45 cycles of denaturation (95 °C for 15 s), annealing (55 °C for 30 s), and extension (72 °C for 30 s). We amplified GAPDH, a housekeeping gene, as a control. The data were analyzed using the sequence detection system software (version 2.2.1, for ABI PRISM 7900HT Software Applied Biosystems or version 2.3 for StepOne Software v2.3, Applied Biosystems) as described in Data Analysis. The software generates the baseline subtracted amplification plot of normalized reporter values (ΔRn) versus cycle number. The amplification threshold was set at 6–7 of the ΔRn linear dynamic range (50–60% of maximum ΔRn). The fractional cycle at which the intersection of the amplification threshold and the plot occurs was defined as the threshold cycle (Ct-value) for the plot. The samples that gave a Ct-value within 45 cycles were considered to be positive for the mRNA expression. Then, quantitative analysis was performed using the ΔΔCT method, as described previously [48].

The forward (F) and reverse (R) primers are as follows (Table 1):

### 4.4. ELISA

ELISA was performed according to manufacturer’s protocol (Quantikine IFN-β ELISA Kit MIFNB0, R&D Systems, Inc., Minneapolis, MN, USA). Briefly, fifty µL of supernatant of the culture was conducted to the ELISA assay. The standard curve was made according to the concentration of recombinant mouse IFN-β and optical density value at the 450 nm of the standards, and then the concentration of IFN-β in each group of supernatants was calculated according to the equation of the standard curve.

### 4.5. Immunocytochemistry

SIM-A9 microglial cells (3.2 × 10^4^) were cultured in poly-DL-lysine coated Lab tek chambers. After the cells were treated as described above (in 5.2), they were fixed in ice-cold MeOH/Acetone (50% MeOH and 50% Acetone) for 5 min at −30 °C. For immunostaining, the cells were blocked with blocking solution (PBS containing 3% bovine serum albumin and 0.02% sodium azide) for 1 h at room temperature (RT). The cells were then incubated in rabbit anti-RTP4 antibody (1:200) (MyBioSource, San Diego, CA, USA) in blocking solution at 4 °C overnight. The cells were rinsed 3 × 5 min with PBS and incubated secondary antibody (1:1000) (Alexa-488-conjugated goat anti-rabbit IgG) (Cell Signaling Technology, Danvers, MA, USA) in blocking solution for 2 h at RT. The cells were washed 3 × 5 min with PBS, mounted on slides, and coverslipped with ProLongTM Diamond Anti-fade Mountant with DAPI (Thermo Fischer Scientific, Waltham, MA, USA). The slides were stored at −30 °C until analysis.

### 4.6. Imaging and Quantification by ImageJ Software

The histochemical analysis of the stained cells (from Section 2.2) were performed on images acquired using a confocal laser scanning microscope (LSM-800) (Carl Zeiss Meditec Co., Ltd., Tokyo, Japan) (40× objective). The signal intensity of RTP4 as the GFP fluorescent was calculated using macros in Image J software (ImageJ bundled with 64-bit Java 8).

### 4.7. Data and Statistical Analysis

The data were expressed as mean ± S.E.M. A difference was considered to be significant at *p* < 0.05. Normal distribution of the data was verified before performing parametric statistical analysis. Wherever appropriate, the data were analyzed using one-way ANOVA, followed by Tukey’s post hoc tests or multiple unpaired t-test with significance set at *p* < 0.05. All the calculations were performed using GraphPad Prism 9 or 10 software (GraphPad Software, Inc., San Diego, CA, USA).

## 5. Conclusions

In this study, we showed that LPS stimulation through TLR4 and IFNR-dependent mechanisms triggers the expression of RTP4 in microglia following treatment with LPS. However, the specific function of RTP4 in the microglial response to LPS remains to be elucidated in future studies.

## Figures and Tables

**Table 1:** The list of primers used in this study. The forward (F) and reverse (R) primers has been shown in this table.

GAPDH-F	TGAAGGTCGGTGTGAACG
GAPDH-R	CAATCTCCACTTTGCCACTG
RTP1-F	TGGAAGCCCAGTGAGAAGC
RTP1-R	AGCAGAAGTTGCAGCCTGAG
RTP2-F	AGCTTTCTGTTCTTCCTTGGG
RTP2-R	GCCACCTCCATCTTCTCGTAG
RTP3-F	TGCAAGAGGTGAAACCCTGG
RTP3-R	AGGACAGTGGAACCTAGCAAAG
RTP4-F	GGAGCCTGCATTTGGATAAG
RTP4-R	GCAGCATCTGGAACACTGG
IL-1β-F	TGTAATGAAAGACGGCACACC
IL-1β-R	TCTTCTTTGGGTATTGCTTGG
TNFα-F	TTGCTCTGTGAAGGGAATGG
TNFα-R	GGCTCTGAGGAGTAGACAATAAAG
iNOS-F	GACGAGACGGATAGGCAGAG
iNOS-R	GTGGGGTTGTTGCTGAACTT
IFN-α-F	AGGACAGGAAGGATTTTGGA
IFN-α-R	GCTGCTGATGGAGGTCATT
IFN-β-F	CATCAACTATAAGCAGCTCCA
IFN-β-R	TTCAAGTGGAGAGCAGTTGAG

## Data Availability

Data is contained within the article and Supplementary Materials.

## Supplementary Materials

The following supporting information can be downloaded at https://www.mdpi.com/article/10.3390/ijms252413676/s1.
