# Supplementary material for: Inflammatory Stimulation Upregulates the Receptor Transporter Protein 4 (RTP4) in SIM-A9 Microglial Cells
Source: Int J Mol Sci. 2024 Dec 21;25(24):13676. doi: 10.3390/ijms252413676 (PMC11728443; doi:10.3390/ijms252413676)
Supplement: Supplementary file 1 [file ijms-25-13676-s001.zip › ijms-3364175-supplementary.pdf]

Inflammatory Stimulation Upregulates the Receptor Transporter Protein 4 (RTP4) in SIM-A9 Microglial Cells.

Wakako Fujita [1,2,*], and Yusuke Kuroiwa [3]
1 Laboratory of Pharmacotherapeutics, Faculty of Pharmacy, Juntendo University, Chiba 279-0013, Japan
2 Department of Medical Pharmacology, Nagasaki University Graduate School of Biomedical Sciences, Nagasaki 852-8523, Japan
3 Department of Pharmacology and Therapeutic Innovation, Course of Pharmaceutical Sciences, Nagasaki University, Nagasaki 852-8521, Japan
* Correspondence: w.fujita.ss@juntendo.ac.jp; Tel.: +81-47-354-3311

Supplementary Figure S1

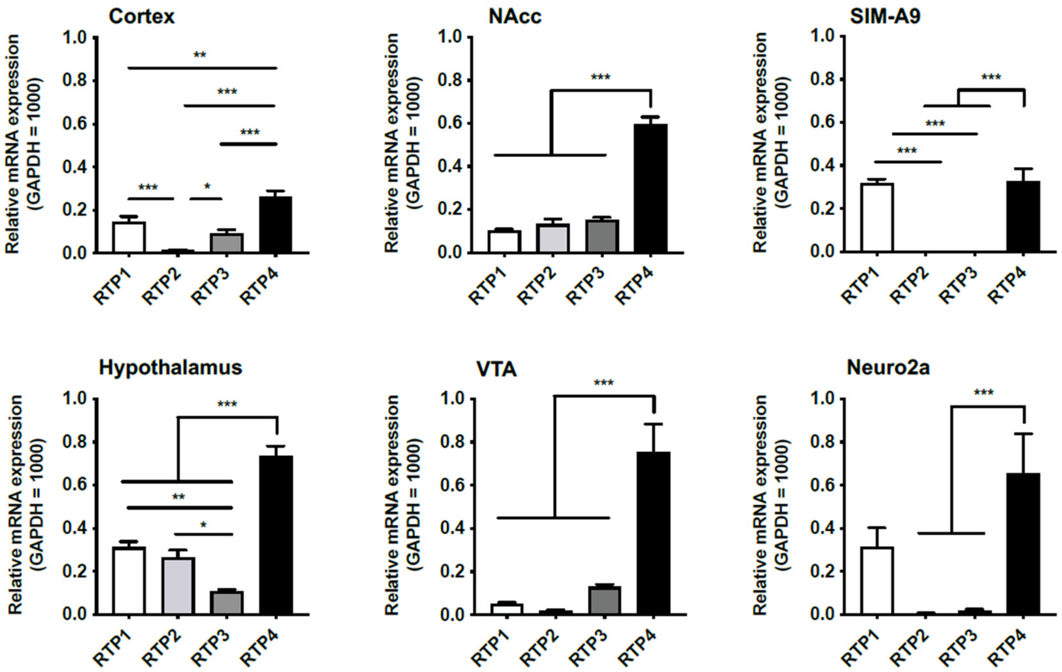

Supplementary Figure S1. Relative mRNA expression of RTP isotypes (*RTP1, 2, 3,* and *4*) in the mouse brain, microglial cell line (SIM-A9), and neuronal cell line (Neuro2a). Data are the mean ± S.E.M. n=6 (Cortex, Hypothalamus, NAcc, VTA), n=7-12 (SIM-A9), n=8-9 (Neuro2a), *p<0.05, **p<0.001, ***p<0.0001, one-way ANOVA and Tukey's multiple comparison test. NAcc: nucleus accumbens, VTA: ventral tegmental area.

Suppl. Methods

Animals. Male C57BL/6 mice (25–35 g; 6–12 weeks old) were obtained from Jackson Laboratories (Bar Harbor, ME, USA). All the mice were maintained on a 12 h light/dark cycle with rodent chow and water available ad libitum and were housed in groups of five until testing. On the test day, the mice were euthanized by cervical dislocation, and various brain regions were collected and transferred into an RNase-free centrifuge tube and stored at -80°C until they were further processed for RTqPCR, as described previously [47]. Animal studies were performed according to the protocols approved by the Institutional Animal Care and Use Committee of the Icahn School of Medicine.

Neuro2a cell culture. Neuro2a cells were grown in complete growth medium (Eagle's Minimum Essential Medium (EMEM) with 10% fetal bovine serum and 1% penicillin–streptomycin (P/S)). The cells were seeded in a 24-well plate (200,000 cells/well) in complete growth medium and incubated overnight at 37°C with 5% CO2. After 48 h of incubation, the cells were washed once with cold phosphate-buffered saline and then collected with 300 μL of cold RLT buffer (QIAGEN, Hilden, Germany). The cell lysates were transferred into RNase-free centrifuge tubes and stored at -80°C until further processing for reverse-transcription quantitative polymerase chain reaction (RT-qPCR).

RT-qPCR. RT-qPCR was performed as described in the main text.

Statistical analysis. Statistical analysis was performed as described in the main text.
